# Uncovering Evidence: Associations between Environmental Contaminants and Disparities in Women’s Health

**DOI:** 10.3390/ijerph19031257

**Published:** 2022-01-23

**Authors:** Jelonia T. Rumph, Victoria R. Stephens, Joanie L. Martin, LaKendria K. Brown, Portia L. Thomas, Ayorinde Cooley, Kevin G. Osteen, Kaylon L. Bruner-Tran

**Affiliations:** 1 Department of Microbiology, Immunology and Physiology, Meharry Medical College, Nashville, TN 37208, USA; jrumph19@email.mmc.edu (J.T.R.); jmartin19@email.mmc.edu (J.L.M.); lbrown19@email.mmc.edu (L.K.B.); pthomas13@email.mmc.edu (P.L.T.); acooley17@email.mmc.edu (A.C.); 2 Women’s Reproductive Health Research Center, Department of Obstetrics and Gynecology, Vanderbilt University School of Medicine, Nashville, TN 37232, USA; victoria.r.stephens@vanderbilt.edu (V.R.S.); Kevin.osteen@vanderbilt.edu (K.G.O.); 3 Department of Pharmacology, Vanderbilt University, Nashville, TN 37232, USA; 4 Department of Pathology, Microbiology and Immunology, Vanderbilt University School of Medicine, Nashville, TN 37232, USA; 5 VA Tennessee Valley Healthcare System, Nashville, TN 37208, USA

**Keywords:** women’s health, environmental contaminants, pollution, health disparities, minorities

## Abstract

Over the years, industrial accidents and military actions have led to unintentional, large-scale, high-dose human exposure to environmental contaminants with endocrine-disrupting action. These historical events, in addition to laboratory studies, suggest that exposure to toxicants such as dioxins and polychlorinated biphenyls negatively impact the reproductive system and likely influence the development of gynecologic diseases. Although high-level exposure to a single toxicant is rare, humans living in industrialized countries are continuously exposed to a complex mixture of manmade and naturally produced endocrine disruptors, including persistent organic pollutants and heavy metals. Since minorities are more likely to live in areas with known environmental contamination; herein, we conducted a literature review to identify potential associations between toxicant exposure and racial disparities in women’s health. Evidence within the literature suggests that the body burden of environmental contaminants, especially in combination with inherent genetic variations, likely contributes to previously observed racial disparities in women’s health conditions such as breast cancer, endometriosis, polycystic ovarian syndrome, uterine fibroids, and premature birth.

## 1. What Are Environmental Contaminants?

An unintended consequence of industrialization has been the release of thousands of synthetic chemicals into our environment without consideration of potential adverse effects on human health [1,2]. Additional sources of environmental contamination include cigarette smoke, wildfires, volcanic eruptions, and the burning of fossil fuels [3,4,5]. As will be discussed below, naturally occurring heavy metals are also considered environmental contaminants when their presence exceeds normal background levels.

### 1.1. Persistent Organic Pollutants, Endocrine Disrupting Chemicals, and Heavy Metals

**Figure 1:**
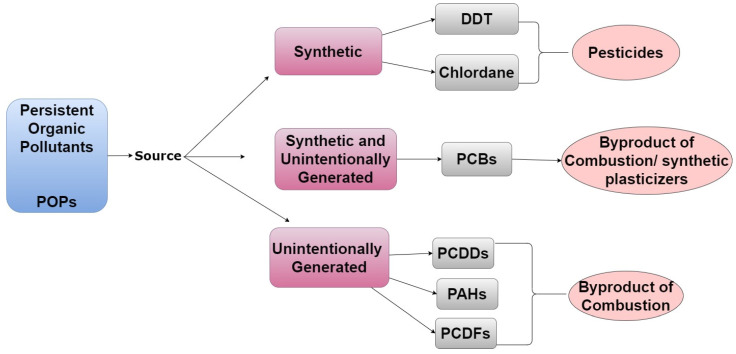
A tree diagram listing emission sources of POPs, representative examples of chemicals from each source, and how/why they are in the environment.

Environmental contaminants generally fall into three categories: persistent organic pollutants (POPs), endocrine-disrupting chemicals (EDCs), and heavy metals. POPs are carbon-based chemicals that are not easily metabolized and exhibit an extended half-life of 10+ years. Because of their ability to bioaccumulate in adipose tissue, they can biomagnify within the food chain; thus, the body burden of toxicants tends to increase with age in both humans and animals [6]. POPs are produced by both natural and anthropogenic processes, though most POPs of concern are produced intentionally for commercial use, as shown in Figure 1 [7].

**Figure 2:**
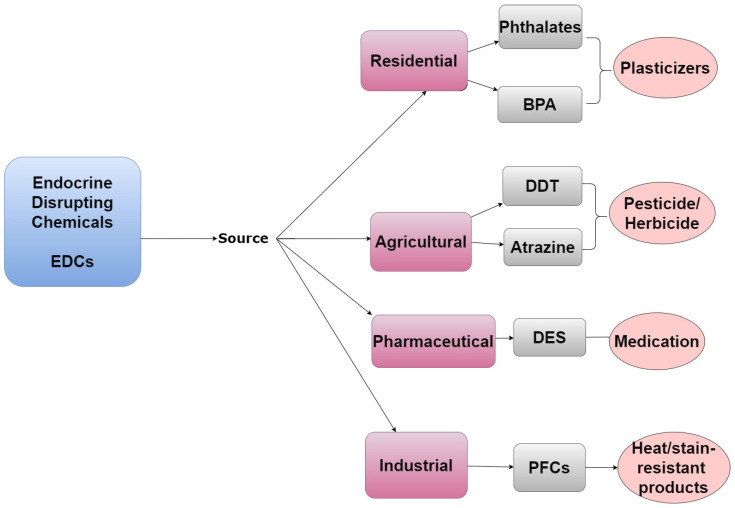
A tree diagram listing the emission sources of EDCs, representative examples of chemicals from each source, and how/why they are in the environment.

Many POPs can disrupt the endocrine system and are therefore also classified as EDCs. The endocrine system is a collection of hormone-secreting glands that are critical to regulating developmental, metabolic, and reproductive processes [5]. EDCs frequently act as hormone agonists or antagonists and influence the endocrine system by activating, blocking, or by altering normal hormone activity via interactions with nuclear receptors or altering metabolism [8]. Steroid hormones and their respective target organs are exquisitely sensitive to interference by EDCs and these chemicals can have wide-ranging effects on human health. Humans can be exposed to EDCs through residential, agricultural, pharmaceutical, and industrial activities, as shown in Figure 2 [9].

**Figure 3:**
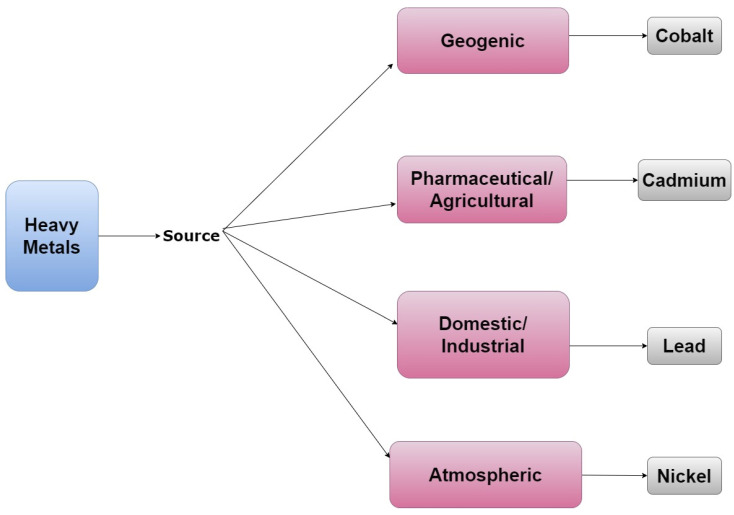
A tree diagram that lists representative examples of heavy metals and their emission sources.

Naturally occurring heavy metals are normally present in the environment as trace elements; however, their accumulation can lead to toxicity—making these compounds environmental contaminants. Although heavy metals are not always classified as EDCs, some have endocrine-disrupting properties. For example, cobalt and cadmium have both been shown to exhibit estrogen-like activity in the absence of estradiol [10,11,12]. Additional metals with endocrine-disrupting properties include arsenic, lead, mercury, chromium, copper, nickel, cadmium, and tin [12,13]. Thus, while heavy metals are naturally occurring and have both geogenic and atmospheric sources their use in industrial, pharmaceutical, and agricultural processes can lead to their excess accumulation in the environment, as demonstrated by Figure 3 [14].

#### 1.1.1. Examples of POPs

Pesticides are well-characterized synthetic POPs. Dichlorodiphenyltrichloroethane (DDT), a pesticide that was widely used in the United States from 1946 to 1972, acts as an estrogen agonist [15]. However, its metabolite, dichlorobiphenyl dichloroethylene (DDE), is an androgen antagonist [16]. Chlordane is another pesticide and POP that was in commercial and residential use in the United States from 1948 to 1988 but was banned due to health concerns. Perfluorinated compounds (PFCs) are examples of POPs that are still used today to create heat-resistant and non-stick kitchenware [17] despite concerns that they can disrupt pregnancy and have been suggested to be carcinogenic [18].

Unintentionally generated POPs include polychlorinated biphenyls (PCBs), polychlorinated dibenzo-p-dioxins (PCDDs), polycyclic aromatic hydrocarbons (PAHs), and polychlorinated dibenzofurans (PCDFs). Although PCBs were previously widely produced for commercial use, intentional manufacturing of these compounds has now been banned in most countries. Nevertheless, they are still released by some industrial processes as well as incineration of household and commercial waste [19]. From the 1950s until 1977, PCBs were synthesized to create microwave ovens, air conditioners, and electric cables [19,20]. PCDDs are byproducts of pesticide manufacture and processes utilizing chlorine bleaching. PCDDs are also produced by volcanic eruptions and forest fires. The generation of PCDFs is associated with the synthesis and incineration of products containing PCBs [21]. Lastly, PAHs are byproducts of cigarette smoke, barbecuing, and grilling [22,23].

#### 1.1.2. Examples of EDCs

Bisphenol A (BPA) is a high-volume EDC with broad residential use. It is used to make plastics for food and beverage storage, and it is a component of epoxy resins that were used to form the lining of food cans and baby bottles in previous years. It was determined that BPA can leach from these and other containers and accumulate in food. Although the use of BPA in food containers has now been banned in most countries, this compound remains in production and can be found in a variety of consumer products. BPA was previously considered to be a weak estrogen; however, more recent studies indicate that this compound exhibits similar potency to estradiol in some cellular contexts [24,25,26]. For this reason, BPA was replaced by bisphenol S (BPS) in most countries in the early 2000s [25,27]. Unfortunately, BPS appears to have similar adverse health effects as those associated with BPA [28]. Phthalates are another widely used residential EDC with a short half-life in humans. They are used to make plastic flexible and more durable. Phthalates are also incorporated into personal care products including soap and hair spray due to their binding and solvent properties [29].

Pharmaceutical EDCs include diethylstilbestrol (DES), a synthetic estrogen that was prescribed to pregnant women to prevent miscarriages and preterm birth from 1938 to 1971 [30]. DES was also injected into livestock to increase meat production [31]. DES was banned as a therapy for pregnant women following the clinical manifestations of its endocrine-disrupting properties. Clinical manifestation of in utero exposure to DES included increased incidence of vaginal cancers in reproductive-age daughters as well as adverse effects in mothers and sons [32]. Shortly after DES was banned as a pharmaceutical, the United States Food and Drug Administration also banned its use in meat production [33].

Agricultural EDCs include the pesticide DDT, a POP described above. Atrazine is an agricultural EDC that currently remains in use despite having been found to influence the female reproductive system by dysregulating the hypothalamic–pituitary–ovarian axis [34]. Industrial EDCs include PCB-153 which was previously used to create dielectric insulating fluid. PCB-153 is no longer synthesized today, but the chemical persists in the environment and is the most prevalent PCB found in the human body [7]. Industrial EDCs also include PFCs, which are also considered POPs.

#### 1.1.3. Examples of Heavy Metals

Cobalt is an example of a geogenic heavy metal, whereas cadmium and copper are considered pharmaceutical compounds [35,36]. Domestic and industrial heavy metals include lead [14] while atmospheric heavy metals include nickel and copper. Cadmium and nickel are also considered agricultural heavy metals [37].

**Figure 4:**
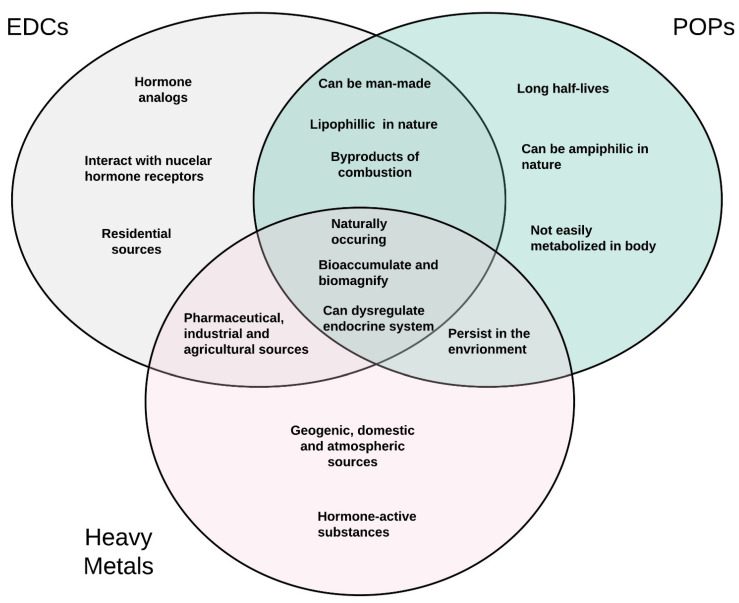
A three-way Venn diagram displaying commonalities and differences in the characteristics of POPs, EDCs, and heavy metals.

Although heavy metals, POPs, and EDCs have overlapping characteristics, each group also has unique distinctions as noted in Figure 4. Members of each of these groups of chemicals have also been shown to influence the endocrine system and are well positioned to influence the development of reproductive diseases in women. Since the literature and environmental justice movements suggest that minorities are more likely to live in areas with known environmental contamination [38,39], herein, we will review the current literature to identify potential associations between environmental contaminant exposure and the development of diseases known to exhibit racial disparities among women.

### 1.2. Risk Factors Associated with Human Exposure to Environmental Contaminants

A plethora of factors influences a person’s risk of exposure to environmental contaminants. These factors include socioeconomic status, occupation, diet, and personal habits. Although race is not a risk factor for environmental exposures per se, certain minority groups are more likely to fall into social groups (e.g., low socioeconomic status) that are at increased risk of exposure.

#### 1.2.1. Socioeconomic Status, Occupation, and Geographic Locale

Previous studies suggest that socioeconomic status—which is correlated to occupation and geographic locale—plays a significant role in the risk of exposure to environmental contaminants [40]. In the United States, low-income residents, which are disproportionally minorities, have a more pronounced exposure to particulate matter-emitting facilities [41,42]. Furthermore, it has been reported that Black and Hispanic women living in Chicago were more likely to reside in areas with higher ambient concentrations of heavy metals including cadmium, mercury, and lead compared to white women [43].

The risk of exposure to environmental contaminants also varies by occupation. United States military personnel, which includes a disproportionally high percent of Black and Hispanic Americans, are at increased risk of exposure to environmental contaminants [44]. Additional occupations that may lead to disproportionate exposure to environmental contaminants include firefighters, miners, farmers, and industrial workers [45]. Furthermore, Ash et al. reported that individuals in low-paying jobs, which are more likely to be Black or Hispanic, were subjected to higher incidences of exposure than their counterparts with higher-paying occupations [46].

Furthermore, low socioeconomic status is positively correlated to high exposure to air pollution globally [47]. A study conducted in Italy supported the correlation between socioeconomic status and atmospheric pollution, reporting that low socioeconomic status increased the risk of exposure to particulate matter and nitrogen dioxide—each of which is a public health concern [48]. Citizens of developing countries are also at heightened risk of exposure to air pollution. However, it is predicted that much of the pollution experienced in developing countries is generated indoors as opposed to industrialized countries where pollution is generated from activities such as fossil fuel burning [49]. For example, in some developing nations such as sub-Saharan Africa, approximately 83% of the population relies on cooking methods that generate indoor pollution (e.g., solid fuel burning). In these settings, women are typically responsible for cooking and are therefore more susceptible to being exposed to this form of indoor pollution [50].

Peruvian and Guatemalan women are susceptible to exposure to PAHs and other harmful compounds through cooking methods as well [51]. Additionally, indoor exposure to benzo(a)pyrene (BaP) from cooking oil fumes has been reported to range from 19 to 23 μg/m^3^ in Taiwan [52]. Similarly, in rural Burundi, indoor BaP emission from wood combustion is estimated to be approximately 100 μg/m^3^ [53]. Socioeconomic status, occupation, and geographic location each influence one’s risk of exposure to environmental contaminants. Unfortunately, persons of color are disproportionally affected by factors that increase the risk of environmental contaminant exposure compared to whites.

#### 1.2.2. Diet

As stated above, POPs, EDCs, and heavy metals have the potential to bioaccumulate and biomagnify within the food chain [54]. Thus, dietary habits can influence the body’s burden of environmental contaminants. Although data tracking dietary environmental contaminant exposure are scarce, a group in India recently reported that meat and dairy products are often traced with environmental contaminants [55]. Law et al. reported that chicken skin and fish from China had higher levels of environmental contaminants compared to beef and pork [56]. Food packaging, which is prevalent in industrialized countries, is also a major source of environmental contaminants because EDCs/POPs can leach into food [57]. Therefore, the food-borne risk of exposure to environmental contaminants may vary depending on the country of origin, food preferences, and food storage practices.

#### 1.2.3. Use of Personal Care Products

Individuals that use personal care and beauty products are also susceptible to excess chemical exposures because these products frequently contain environmental contaminants [58]. Zota et al. reported that chemical exposures from beauty products vary by race and Black women were more likely to use products that contain EDCs. The group reported that Black women were at risk of being exposed to heavy metals, parabens, and phthalates through beauty products such as hair relaxers and vaginal douches [59]. Using a nationally representative sample of reproductive-aged women, Branch et al. found that Black women in the United States douched more frequently than other races. The group also found that women who douched had a 150% higher exposure to diethyl phthalate [60]. Studies suggest that hair care products used by Black women are more likely to contain placental-derived extracts, typically from cows or sheep, which have endocrine-disrupting activity. James-Todd et al. reported 49.4% (African American) and 26.4% (African-Caribbean) of Black women used hair products that contain placental extracts compared to 7.7% of white women [61].

The use of hair products that contain placental extracts or other EDCs among Black women has also been linked to altered reproductive development. Tiwary et al. reported that the use of hair products containing placental extracts on Black daughters between the ages of 14 and 93 months led to the premature development of breast and pubic hair. However, after the use of these products ceased, their sexual development regressed [62]. The group also reported that among military personnel, non-whites were four times more likely to use hair products containing EDCs and placental extracts compared to whites. However, women of all races/ethnicities were more likely to use these products compared to men [63]. Together, these studies suggest that the environmental contaminants found in hair products can negatively impact the endocrine system, potentially contributing to racial health disparities and sex-related differences in disease occurrence [59,62,63,64].

## 2. Scope of Review

Overall, numerous groups have reported that women of color exhibit a higher body burden of environmental contaminants [65,66,67]. Since many environmental contaminant exposures have been linked to reproductive dysfunction, we reviewed the literature to address the question: Does environmental contaminant exposure contribute to racial disparities in women’s health? We will discuss the potential role of environmental contaminants in the development of breast cancer, endometriosis, fibroids, polycystic ovarian syndrome, and premature birth. We chose to investigate the correlation between toxicant exposure and these women’s health conditions because many have higher incidences (fibroids, PCOS, and premature birth) and/or mortality rates (breast cancer) in women of color. We chose to investigate the correlation between toxicant exposure and endometriosis because, historically, women of color have been less likely receive adequate diagnoses. It should be noted that in this review, the terms Black, Hispanic, Asian, and white encompass multiple ethnicities. Research articles cited were selected by searching key terms such as “Black women” and “toxicants”/“pollution” and “endometriosis” using the PUBMED library.

## 3. Breast Cancer

Breast cancer, the uncontrolled proliferation of breast cells, frequently metastasizes to other areas of the body. Breast cancer affects women across all races and approximately 1 in 10 women have been diagnosed with breast cancer worldwide. Breast cancer is highly influenced by the endocrine system and steroid hormone response. Approximately 70–75% of breast carcinomas express estrogen and progesterone receptors and are considered luminal tumors [68]. Luminal tumors are further classified as luminal A or B. Luminal A tumors express estrogen and progesterone receptors, whereas luminal B tumors typically exhibit reduced expression of these receptors along with increased expression of HER2 (human epidermal growth factor receptor 2) [69]. HER2 is a proto-oncogene normally present on epithelial cells of the breast; however, cancer cells with a higher-than-normal level of expression of HER2 are considered HER2 positive and are typically the most aggressive subtype [70]. Breast carcinomas that are deficient in estrogen receptors, progesterone receptors, and HER2 are termed triple-negative breast cancer. Importantly, since breast cancer treatment typically targets these receptors, triple-negative cancers are more difficult to treat compared to other subtypes [71].

Although the occurrence of breast cancer does not exhibit significant racial disparity, recent studies suggest that there are clear disparities regarding the type and aggressiveness of breast cancer that women of color develop compared to white women. Importantly, in the United States, survival rates are significantly impacted by both the type of breast cancer and its stage at the time of diagnosis. Black women are typically diagnosed with breast cancer at a younger age (59 years old) compared to white women (63 years old). Despite the earlier diagnosis, Black women are more likely to develop triple-negative and/or metastatic breast cancer when compared to non-Hispanic white women [72]. For poorly understood reasons, the incidence of breast cancer among Black women has increased 0.4% per year since 1975 but has remained stagnant among white women [72].

It is well known that genetic variations also contribute to an individual woman’s risk of developing breast cancer. Women with BRCA1/2 mutations are at increased risk of breast cancer and are known to contribute to the familial occurrence of this disease in most, if not all, ethnicities [73,74]. Beverly et al. reported that white women with breast cancer exhibited a higher prevalence of estrogen and progesterone receptors compared to Black women with breast cancer—which may contribute to improved survival rates in white women since many breast cancer treatments target hormone receptors [75]. An additional study found that two single-nucleotide polymorphisms (SNPs) (rs590688 and rs10895054) in the progesterone receptor gene were significantly associated with breast cancer in Black women, but not in white women [76].

Lifestyle habits may also influence disparities in breast cancer. For example, it has been reported that cigarette smoking (an activity that releases PCDDs) promotes the metastasis of breast cancer into the pulmonary system [77,78]. Despite these data and the known influence of inherent genetic differences, the potential role of gene–environment interactions in the development of cancer and/or the racial disparity in breast cancer type and mortality has not been well investigated. Nevertheless, Lambda et al. described a wide variation in the expression of CYP3A enzymes across racial groups as a consequence of gene polymorphisms and/or isoform expression [79]. Cytochrome P450 monooxygenases (CYPs) play an important role in the metabolism of xenobiotics, such as environmental contaminants; thus, racial differences in expression of one or more of these genes could readily play a role in increasing or decreasing cellular response to environmental contaminants, thereby influencing disease pathogenesis.

The potential for gene–environment interactions is relevant to the discussion regarding EDC exposure and breast cancer, as PCDDs and some PCBs appear to promote breast cancer metastasis into the lymph node [78]. Reynolds et al. collected adipose tissue from breast cancer patients of various races and measured the levels of environmental contaminants. The group found that Black, Asian and Hispanic women exhibited significantly higher concentrations of 12378-PeCDD and 123478-HxCDF compared to white women [80]. A separate study reported that Black women exhibited mean total pesticide and PCB concentrations that were 10% higher than white women. The group also reported that increased adipose PCB concentrations were associated with reoccurring tumors [81].

A longitudinal cohort study compared contaminant body burden and mortality rate in white and Black women diagnosed with invasive breast cancer between 1993 and 1996. White participants had a mean total lipid PCB concentration of 0.38 µg/g, and Black participants had a mean of 0.56 µg/g. Black women were also more likely to have more aggressive forms of breast cancer, resulting in 61% of Black women versus 46% of white women succumbing to disease over the course of the study. Increasing body burdens of PCB-74, 99, and 118 and total PCBs were associated with a 33–40% increase in breast cancer mortality, with the strongest effects within 5 years of diagnosis. Black women were more likely to have a higher body burden of PCB-74, which was associated with a larger elevated risk of 5 year breast cancer-specific mortality compared to white women [82].

Exposure to heavy metals has also been linked to the development and progression of breast cancer [83]. White et al. reported that exposure to heavy metals was associated with increased breast density, which is strongly related to the risk of breast cancer [84]. Kaushiva et al. reported that Black and Hispanic women living in census tracts with high quartile ambient concentrations of heavy metals exhibited an increased risk of breast cancer compared to women living in low quartile environments. This higher incidence of breast cancer was associated with exposure to cadmium, lead, and nickel [43]. Overall, the current literature suggests that environmental exposures, combined with genetic variations, likely contribute to the previously observed racial differences in the type of breast cancer, disease recurrence, and rate of mortality.

## 4. Endometriosis and Endometriosis-Related Infertility

Endometriosis is defined as the growth of endometrial tissue at an extra-uterine site and is characterized by altered hormone responsiveness, chronic pelvic pain, and, frequently, infertility [85]. The term endometriosis was coined in 1921 by John A. Sampson, who later proposed the landmark theory of retrograde menstruation as its cause in 1927 [5,86]. In 1938, Joseph Meigs noted that endometriosis was more commonly diagnosed in white, affluent women and theorized that the disease was rare in Black women [87]. In the early and mid-1900s, reports continued to suggest that the prevalence of endometriosis was doubled in white women compared to Black women and rarely considered access to care as a possible confounder. In the 1970s, studies conducted by Donald L. Chatman demonstrated that more than 20% of Black women who had endometriosis were misdiagnosed with pelvic inflammatory disease; he, therefore, concluded that the incidence of disease was similar between races [87].

We now know that endometriosis affects approximately 10% of women worldwide and that all races/ethnicities can be impacted. However, it remains unclear if there are disparities in disease prevalence between women of different ethnicities. A recent report suggested that Black women are still less likely to be diagnosed with endometriosis than white women and highlight a likely continuing belief that Black women are at lower risk of disease [88]. This paper also suggested the possibility of heterogeneity in endometriosis phenotype or clinical presentation between racial/ethnic groups which may impede diagnosis. Furthermore, numerous studies support a link between EDC exposure and the development of endometriosis. Since minority populations are more likely to exhibit exposure to such compounds, it is interesting to speculate whether the body burden of EDCs could contribute to heterogeneity in disease presentation.

A link between EDCs and endometriosis was first identified following the administration of DES as a therapeutic agent for pregnant women to prevent early pregnancy loss and preterm birth. Although the drug had no impact on pregnancy outcomes, daughters exposed to DES in utero were at increased risk for developing endometriosis and infertility later in life [89]. A chemical plant explosion in Seveso, Italy in 1976 resulted in the release of a toxic cloud containing high levels of 2,3,7,8-tetrachlorodibenzo-p-dioxin (TCDD, commonly known as dioxin) [90]. The population of Seveso and surrounding areas have been carefully monitored since the explosion, revealing a modestly increased risk in endometriosis in women with sera levels of 100 parts per trillion (ppt) or higher of TCDD [91,92]. For comparison, the current estimated “background” body burden of TCDD in industrialized countries is 2 ppt [93].

Additional studies found that PCB-138, 153, and 180, as well as non-estrogenic PCBs, were found in high concentrations in women with endometriosis [94]. A relationship between EDCs and endometriosis is also supported by studies using animal models. For example, Rier et al. identified endometriosis in a primate colony exposed to dietary TCDD. Animals with the most severe disease were those with the greatest exposure to TCDD [95].

Exposure to heavy metals may also be linked to the development of endometriosis as well as infertility. Jackson et al. reported a dose-dependent association between cadmium and endometriosis [11]. A separate group found that increased blood concentrations of lead, but not cadmium, were associated with infertility in Taiwanese women [96]; however, this group did not examine the relationship between endometriosis and heavy metals.

Although Black women have been reported to have higher body burdens of environmental contaminants that have been linked to the development of endometriosis [97]; to date, only limited studies have examined the relationship between contaminant body burden and endometriosis between races. However, differences in steroid synthesis and metabolism have been observed between Black and white women and thus may influence response to EDCs. For example, epidemiological and experimental studies indicate that luteinizing granulosa cells are dysregulated in endometriosis and endometriosis-related infertility [98,99,100], leading to altered production of aromatase. Aromatase is the enzyme that converts androgens to estradiol and is essential for the ovarian synthesis of this steroid. Endometriosis is an estrogen-dependent disease and inhibitors of aromatase have been investigated as therapeutic agents [101]. Shaw et al. reported that Black women exhibited increased ovarian aromatase mRNA expression, as well as increased estradiol levels and reduced androgen to estrogen ratios in their follicular fluid compared to white women [102]. The group suggested that this disparity was the result of a genetic variation in Cytochrome P450 19 (CYP19), the gene that encodes aromatase [102,103]. Notably, modulation of steroidogenic enzymes such as aromatase is an important mechanism of action for estrogenic EDCs.

Thus, as a consequence of CYP19 gene variants, Black women may exhibit heightened expression of aromatase following environmental exposures. Although several studies have explored gene–environment interactions in the risk of endometriosis [104,105], to our knowledge, these have not included a discussion on racial differences. Hence, women of color may have genotypes and phenotypes that make them susceptible to endometriosis, but misconceptions regarding risk combined with years of deficits in diagnostic measures may contribute to the reduced identification of disease among this group.

## 5. Polycystic Ovarian Syndrome

Polycystic ovarian syndrome (PCOS) is defined as a common hereditary endocrinopathy affecting 4–6% of reproductive-age women [106,107]. PCOS is associated with menstrual dysfunction, infertility, hirsutism, acne, obesity, and metabolic syndrome. It is classified into four phenotypes: Phenotype A is most severe and includes hyperandrogenism, ovulatory dysfunction, and polycystic ovaries; Phenotype B includes hyperandrogenism and ovulatory dysfunction; Phenotype C, known as ovulatory PCOS, includes hyperandrogenism and polycystic ovaries; Phenotype D, also known as non-hyperandrogenic PCOS, includes ovulatory dysfunction and polycystic ovaries [108].

Epidemiology studies suggest that women of color exhibit an increased risk of developing PCOS compared to white women [106,109,110]. Based on an assessment of symptoms, Goodarzi et al. reported a PCOS prevalence of 13% among Hispanic women, a rate more than twice that of the general population [111]. However, other studies failed to corroborate a significant increase in the prevalence of PCOS among this population compared to other groups [112,113]. A separate study compared the development of PCOS across multiple ethnicities finding the lowest incidence among Chinese women and a similar incidence between white, Black, and Middle Eastern women [110]. This study also highlighted the importance of utilizing the same diagnostic criteria when comparing different studies as comparing prevalence rates using different methods resulted in the appearance of racial disparity. Engmann et al. assessed 702 women with PCOS for phenotypic variations across several racial categories. Since all women in the study were previously diagnosed with PCOS, they were unable to address the disease prevalence between racial groups; nevertheless, they found interesting phenotypic variations. They found that Hispanic women tended to have the most severe phenotype of PCOS (presenting with both hyperandrogenism and metabolic syndrome) while Black women displayed the mildest phenotype compared to other racial groups [114].

The ethnic variations observed in PCOS phenotype and clinical symptoms may be related to genetic and/or environmental exposure differences. Additionally, the potential importance of interactions between genotype and the environment must also be considered [112]. Indeed, increasing evidence suggests that environmental contaminants play a role in the development of PCOS. Takeuchi and Kandaraki et al. reported that serum BPA levels were higher in hyperandrogenic women with PCOS compared to non-hyperandrogenic women with PCOS and healthy controls [115,116]. A separate group reported that increases in serum BPA levels were positively correlated with serum testosterone levels in women with PCOS compared to healthy women [117]. Vagi et al. conducted a case–control study to determine the correlation between various environmental contaminants and PCOS. The group reported that women with PCOS had significantly higher serum concentrations of perfluorooctanoate and perfluorooctane sulfonate. They also reported that women were up to 7.5 times more likely to have PCOS if they had detectable levels of PCB-153, 170, 180, 183, or 196 and 203 [118]. Furthermore, the group reported that there was a negative correlation between phthalate body burden and PCOS. More specifically, women with PCOS had lower urinary concentrations of mono benzyl phthalate (mBzP) [118], suggesting poor metabolism of xenobiotics. Animal models also support a link between PCOS and environmental contaminants. Recent reports suggest that the direct exposure of pregnant rats to either vinclozolin (a widely used fungicide) or DDT (an insecticide now banned in most countries) was linked to the development of ovarian changes consistent with PCOS in three subsequent generations via epigenetic processes [119,120].

Heavy metal exposure also influences the development and progression of PCOS and may be associated with metabolic symptoms of the disease. Kurdoglu et al. reported that women with PCOS exhibited higher serum levels of copper and zinc, but lower levels of lead compared to healthy women [121]. However, Kirmizi et al. reported that women with PCOS exhibited higher levels of cadmium, lead, and mercury and lower levels of zinc and copper [122]. As previously suggested by Wang and Alvero et al., it seems likely that individual and or ethnic variations in genotype combined with environmental exposures ultimately determine a woman’s overall risk of PCOS [112].

## 6. Uterine Fibroids

Uterine leiomyomas, more commonly known as fibroids, are the most prevalent benign smooth muscle neoplasm of the female reproductive system, with up to 70% of women developing this disease by menopause [123,124,125,126]. Clinical evidence suggests that fibroids are hormone dependent since they rarely occur before menarche and shrink substantially after menopause [124,127]. Additionally, fibroid tumors exhibit increased expression of estrogen receptor-α gene and protein compared to healthy, surrounding tissues [128,129,130]. Although most women with fibroids are asymptomatic, the symptomatic disease can be a major source of morbidity and have a substantial adverse impact on a woman’s quality of life. Symptoms of uterine fibroids include pelvic pain and uterine bleeding. The symptoms can be so severe that they cause infertility and lead to 33–50% of all hysterectomies [123,125].

Black women have higher incidences of fibroids at younger ages and have more severe symptoms compared to white women, suggesting that racial differences contribute to disease pathogenesis [123,124]. Wise et al. reported that the use of hair relaxers among Black women increased their risk for uterine fibroids and that this risk was further increased in women who relaxed their hair frequently [131]. Hair relaxers and other personal care products frequently used by Black women have been demonstrated to contain a wide variety of compounds with endocrine-disrupting activity and may be associated with disease risk [132]. Thus, in addition to endogenous estrogen, fibroid growth may also be promoted by exogenous estrogen sources associated with environmental exposures [123,124].

Two longitudinal cohort studies reported that prenatal exposure to DES is associated with the development of uterine fibroids [133,134]; and increased the risk of uterine fibroids by 13% in women over the age of 35 [134]. Trabert et al. reported that women diagnosed with fibroids also exhibited a higher mean concentration of DDT, its metabolite DDE, and PCB-180 in their omental fat. Interestingly, this group also reported that body burdens of contaminants differed between women diagnosed with both uterine fibroids and endometriosis compared to women only diagnosed with fibroids. Women with both diseases had higher body burdens (determined by omental fat) of PCB 99, 138, 146, 153, 196, and 206 compared to women with only endometriosis [135]. Exposure to phthalates has also been reported to influence the development of uterine fibroids. Zota et al. reported that exposure to phthalates was ubiquitous among pre-menopausal women with fibroids; however, Black women with uterine fibroids had levels of specific phthalates (MiBP, MBzP, and MEP) that were 30% higher than white and Hispanic women [136]. The group also reported that differences in phthalates (and their metabolites) may be associated with differences in fibroid size [136].

Qin et al. monitored POP, EDC, and heavy metal levels in Asian women diagnosed with uterine fibroids. The group found that women with fibroids displayed significantly higher concentrations of arsenic, cadmium, lead, and mercury in their subcutaneous fat compared to women without fibroids. They also reported that women with uterine fibroids exhibited higher mean concentrations of various POPs including PCB-126 and 191 [137]. Taken together, current data support a role for exposure to environmental contaminants in the development of uterine fibroids. Furthermore, exposure to environmental contaminants may influence previously observed racial disparities in the development of these benign tumors.

## 7. Premature Birth

Preterm birth (PTB) is a global problem, impacting more than 10% of all pregnancies. Multiple maternal factors, including age, lifestyle choices (cigarette or alcohol use), occupation, and lack of prenatal care are known to contribute to the risk of PTB; however, women with no known risk factors can also deliver early. The normal gestation length of human pregnancy is 40 weeks. Infants that are born preterm (before 37 weeks gestation) or very preterm (before 32 weeks gestation) are at significantly increased risk of death before age 5 compared to babies born at term [138]. For poorly understood reasons, non-Hispanic Black women and Hispanic women are at significantly greater risk of delivering preterm compared to all other racial and ethnic groups [138].

Relevant to the current discussion, numerous studies report an increased risk of PTB following maternal exposure to a variety of manmade toxicants as well as selected heavy metals (reviewed in [139,140]). Although the mechanisms of action of toxicants vary, EDCs that act either directly or indirectly to interfere with progesterone action are particularly well positioned to disrupt pregnancy since the appropriate response of the endometrium and placenta to this steroid is critical to maintaining uterine quiescence until parturition. For example, cadmium, a heavy metal present in tobacco smoke is known to disrupt progesterone production by the ovary and placenta [141,142]. Not surprisingly, numerous studies have linked cadmium exposure to adverse pregnancy outcomes, including PTB. Wu et al. conducted a meta-analysis of 59 different studies and concluded that exposure to heavy metals (cadmium, lead, chromium, copper, magnesium), as well as specific phthalates, were associated with an increased risk of delivering preterm [140]. Interestingly, this study did not reveal an increased risk of PTB in association with organochlorines or PCBs. In contrast, Kofoed et al. found an increased risk of PTB in women exposed to PCBs with lower chlorine content either before pregnancy or during the first trimester [143,144]. In a study from the French West Indies, maternal exposure to chlordecone, an organochlorine pesticide, was associated with decreased gestation length and increased risk of PTB [144].

A major source of environmental contamination is associated with waste incineration, which produces a variety of EDCs, including TCDD, other dioxins, and BaP. Two separate studies conducted in Italy reported an increased risk for PTB in women living near a municipal solid waste incinerator [142,145]. However, a similar study conducted in the United Kingdom found no excess risk of stillbirth, PTB, or infant mortality in association with exposure to incineration-derived particulate matter [146]. These differences may reflect inherent differences within the populations under investigation or different emission standards between countries.

As described previously numerous personal care products have been found to contain compounds with endocrine-disrupting properties. Preston et al. conducted a pilot study examining the use of selected hair products by women and their subsequent incidence of PTB. This study revealed an increased risk of PTB in women that frequently used hair oils, which contain EDCs, during the last trimester of pregnancy [147]. Interestingly, Black women are more likely than other races to report using hair oil [148]. Although data are limited concerning the potential role of EDCs contributing to the known racial disparity in the risk of PTB, some important inferences can be drawn. Minorities, in addition to being more likely to use personal care products containing EDCs, are also more likely to reside in areas contaminated with environmental pollutants [149,150,151,152]. Thus, these and other environmental exposures may contribute to the well-known increased risk of PTB among Black women. Interestingly, although Blacks and Hispanics make up only 12% and 16% of the general U.S. population, respectively, collectively they represent nearly half of the active-duty women in the American armed forces today (29% Black; 20% Hispanic) [153]. Military service is frequently associated with significant exposure to environmental toxicants [44,154] and thus may disproportionately impact women of color and their pregnancies. A recent study suggested that Black women in the United States military are more likely to deliver prematurely compared to their counterparts, regardless of equal access to health care services [155].

Equally important, although pregnancy is largely considered a woman’s health issue, it is well established that the father can also influence pregnancy outcomes and child health (reviewed in [156]). The father’s contribution to pregnancy is conveyed primarily via the sperm, which is known to influence the placental phenotype [21,22,23]. The placenta plays a critical role throughout pregnancy [157,158,159] and placental dysfunction is associated with numerous adverse outcomes including PTB [160,161,162]. Not surprisingly, epidemiological studies suggest that paternal factors, such as obesity and race, can influence pregnancy outcomes in their partners [163,164,165]. Thus, paternal EDC exposure also likely contributes to the overall incidence of PTB [156].

Twin studies have consistently reported a significant familial trend in the incidence of PTB, suggesting that parental genetics may contribute to its incidence [166]. Although most studies focus on maternal genes, at least one study identified the heritability of PTB from the paternal parent [167]. As detailed above, numerous studies support exposure to a wide variety of EDCs in the risk of PTB [139,168,169,170]. Therefore, it is appropriate to consider the potential that gene–environment interactions also contribute to this outcome. However, to our knowledge, few studies have attempted to examine this potential relationship. Nevertheless, since minorities are more likely to reside in areas contaminated with environmental pollutants [149,150,151,152], gene–environment interactions could be a plausible contributor to the racial and geographic disparity of PTB. This line of inquiry is supported by a recent study that examined the presence of specific single-nucleotide polymorphisms in the mother or fetus and the risk of PTB in association with maternal infections [171]. They found a significant association with six different SNPs, PTB, and a recent maternal history of a vaginal infection or urinary tract infection. These data demonstrate that an individual’s genetic make-up, combined with an environmental threat (e.g., infection), can influence the timing of birth. Concerning EDC exposure, Mustafa and colleagues reported that women delivering preterm exhibited higher serum levels of organochlorine pesticides compared to women delivering at term [172]. Furthermore, this risk was enhanced in women that also exhibited the CYP1A1m2 or the GSTM1 null genotypes.

The epidemiology studies described herein have value, but differences in study design, control groups, and timing of exposures contribute to the significant heterogeneity of the findings and limit our ability to draw major conclusions. Additionally, depending on the country of origin, racial and ethnic minorities may be over- or underrepresented within a given study. Thus, while a large body of evidence supports a role for maternal and paternal EDC exposure in contributing to the racial disparity of PTB, finite conclusions cannot yet be drawn.

## 8. Conclusions

This review highlights the current understanding of the potential relationship between exposure to environmental contaminants and disparities in women’s health. The literature suggests that exposure to environmental contaminants increases the risk of breast cancer, endometriosis, PCOS, uterine fibroids, and premature birth in women, as demonstrated by Table 1. Reports also suggest that Black women have higher body burdens of environmental contaminants compared to white women and this disproportionate exposure may be linked to socioeconomic status, geographic location, occupation, dietary habits, and choice of personal care products. Epidemiology also suggests that increased exposure to environmental contaminants in Black women may make this group more susceptible to breast cancer and uterine fibroids. Although numerous studies support a contribution of EDC exposure and the development of endometriosis, PTB, and PCOS, there is a lack of studies that additionally investigate the association of race and environmental exposures. It should be noted that racial disparities in disease diagnosis could also contribute to this deficit in information. It is also worth mentioning that transgenerational mechanisms associated with some of the diseases discussed (endometriosis, PCOS and premature birth) could contribute to a woman’s susceptibility to disease. Therefore, the toxicant exposure history of a woman’s mother or father may increase her susceptibility to disease; however, this may not directly correlate to the woman’s toxicant body burden at the time of diagnosis. It should be noted that this ancestral history of toxicant exposure in combination with the woman’s exposure history throughout life may influence disease development and severity. Nevertheless, the disproportionate exposure of women of color to EDCs, POPs, and heavy metals supports an influence of these compounds in the racial disparity of women’s health conditions.

## Figures and Tables

**Table 1:** List of POPs, EDCs, and heavy metals associated with breast cancer, endometriosis, polycystic ovarian syndrome, uterine fibroids, and preterm birth.

Women’s Health Condition	Associated EDCs/POPs	Associated Heavy Metals	References
Breast Cancer	1,2,3,7,8-PeDCC; 1,2,3,4,7,8-HxCDF; PCB-74; PCB-99; PCB-118	Cadmium; lead; nickel	Reynolds et al. [80]; Muscat et al. [81]; Parada et al. [82]; Kaushiva et al. [43]
Endometriosis	PCB-138; PCB-153; PCB-180; TCDD; DES	Cadmium	Stillman et al. [90]; Homberger et al. [91]; Potera et al. [95]; Lei et al. [97]; Jackson et al. [11].
Polycystic Ovarian Syndrome	BPA; perfluorooctanoate, perfluorooctane; PCB-153; PCB-170; PCB-180; PCB-183; PCB-196; PCB-203	Cadmium; copper; lead; mercury; zinc	Takeuchi et al. [116]; Kandaraki [117]; Konieczna et al. [118]; Vagi et al. [119]; Kurdoglu et al. [122]; Kirmizi et al. [123]
Uterine Fibroids	DES; DDT; DDE; PCB-126; PCB-180; PCB-191; MiBP; MBzP; MEP	Arsenic; cadmium; lead; mercury	Mahalingaiah et al. [134]; Baird et al. [135]; Trabert et al. [136]; Zota et al. [137]; Qin et al. [138]
Endometriosis + Uterine Fibroids	PCB-99; PCB-138; PCB-146; PCB-153; PCB-196; PCB-206	Unknown	Trabert et al. [136]
Preterm Birth	TCDD; BAP; unspecified PCBs (low chlorine content); Chlordecone	Cadmium; lead; chromium; copper; magnesium	Wu et al. [141]; Candela et al. [143]; Kofoed et al. [144]; Kadhel et al. [145]; Santoro et al. [146]

## Data Availability

Not applicable.
